# Supplementary material for: Effects of the Ketogenic Diet on Microbiota Composition and Short-Chain Fatty Acids in Women with Overweight/Obesity
Source: Nutrients. 2024 Dec 19;16(24):4374. doi: 10.3390/nu16244374 (PMC11679786; doi:10.3390/nu16244374)
Supplement: Supplementary file 1 [file nutrients-16-04374-s001.zip › Supplementary File S1.pdf]

Supplementary File S1

Example of Ketogenic Diet Menu

Breakfast

- Omelette with 2 eggs + 1 slice of old cheddar + 2 tablespoons of oil
- 5 olives
- 3 whole walnuts
- Plenty of greens + cucumber (without oil)

Lunch

- Vegetable dish (mushrooms, zucchini, spinach, purslane, cauliflower, broccoli one or more) + 1 tbsp oil
- 2 tbsp strained yoghurt
- Salad + 1 tbsp oil

Snack

- 3 whole walnuts
- 2 cucumber

Dinner

- 2 days a week 200 g fish + green salad + 2 tbsp oil
- 2 days a week 150g red meat + green salad + 1 tablespoon of olive oil
- 3 days a week 150 g chicken breast + green salad + 1 tbsp olive oil
