# Supplementary figures and images for: Effects of the Ketogenic Diet on Microbiota Composition and Short-Chain Fatty Acids in Women with Overweight/Obesity
Source: Nutrients. 2024 Dec 19;16(24):4374. doi: 10.3390/nu16244374 (PMC11679786; doi:10.3390/nu16244374)

Supplementary Figure S1

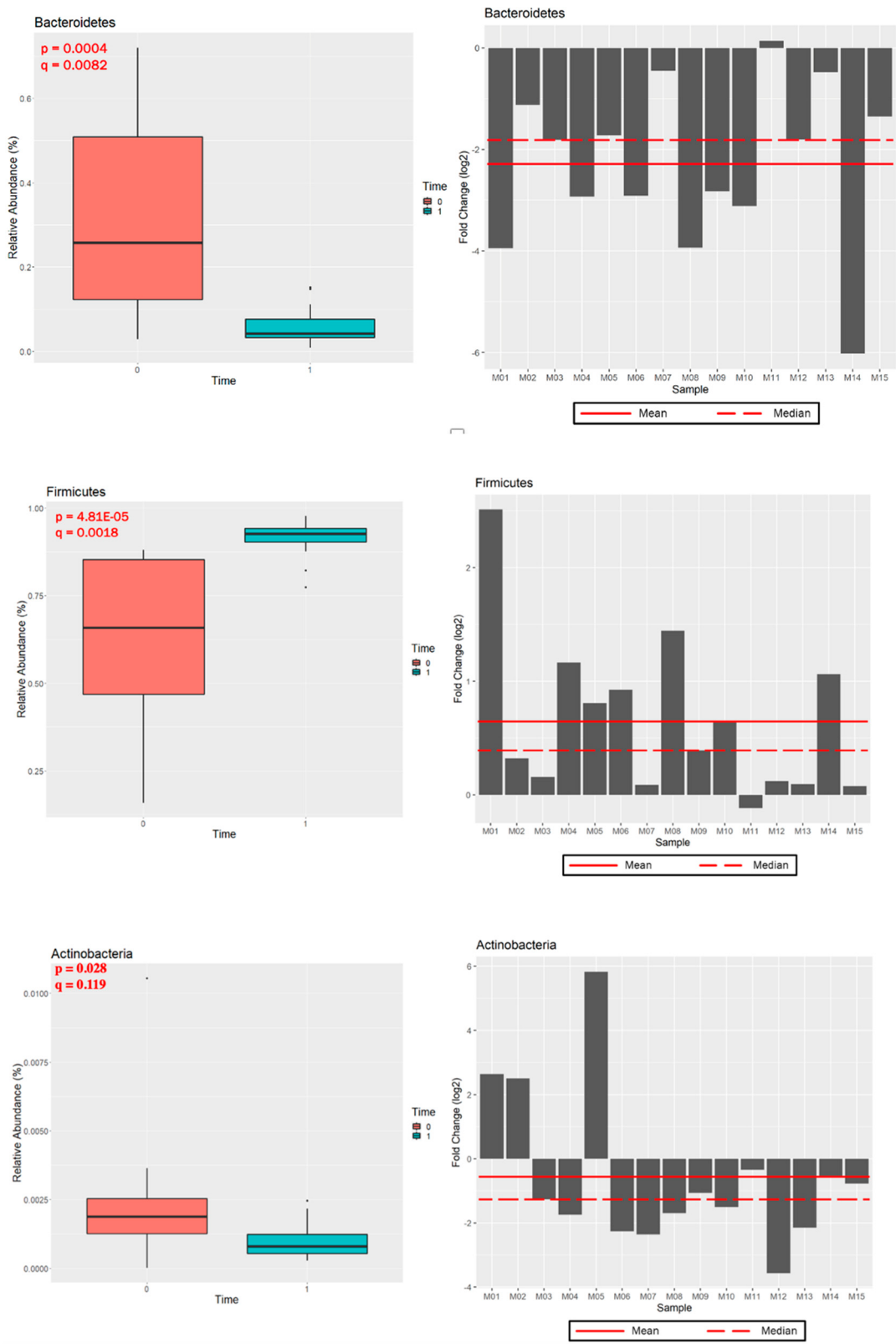

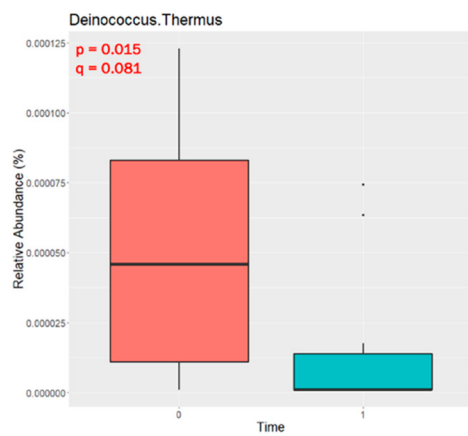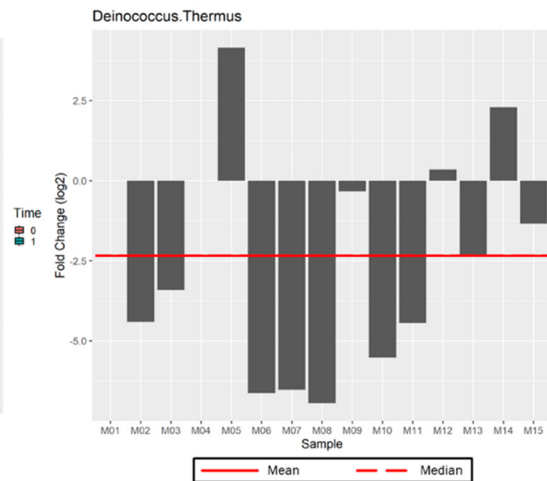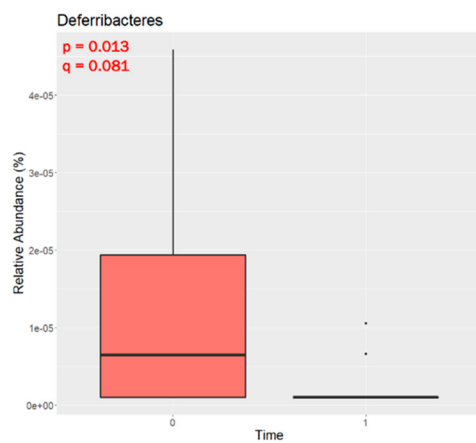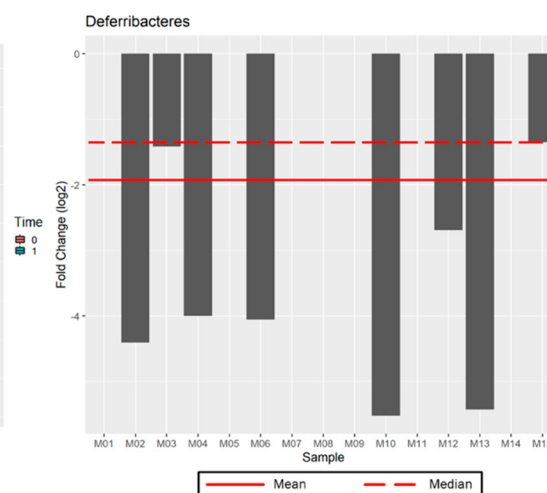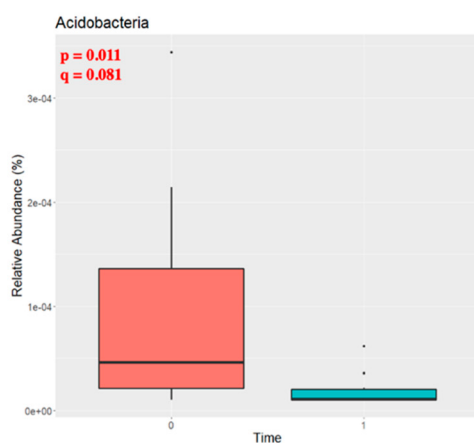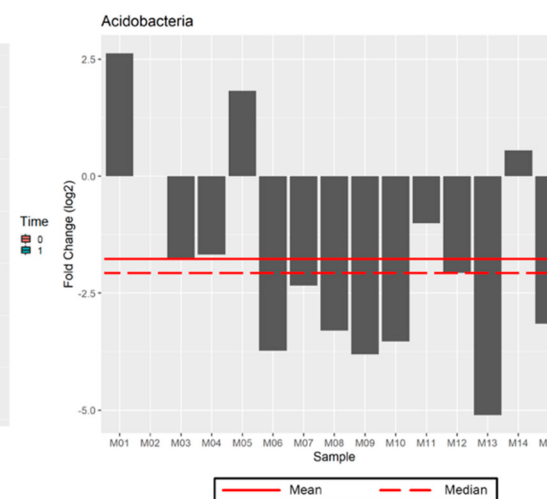

**Supplementary Figure S1.** Phylum with 2-fold or more variation

Supplement: Supplementary file 1 [file nutrients-16-04374-s001.zip › Supplementary Figure S1.pdf]

Supplementary Figure S2

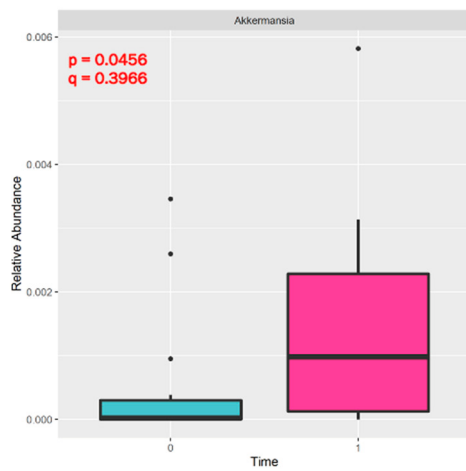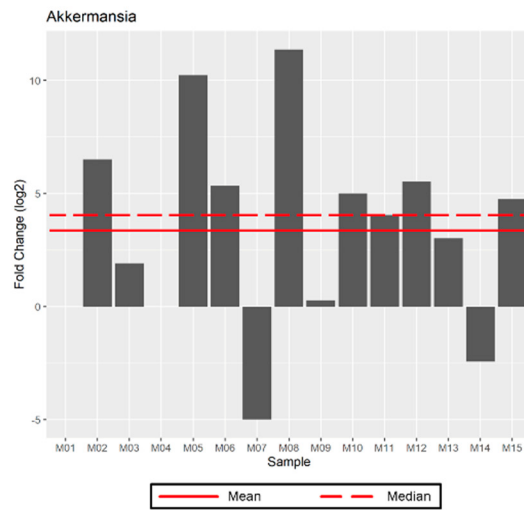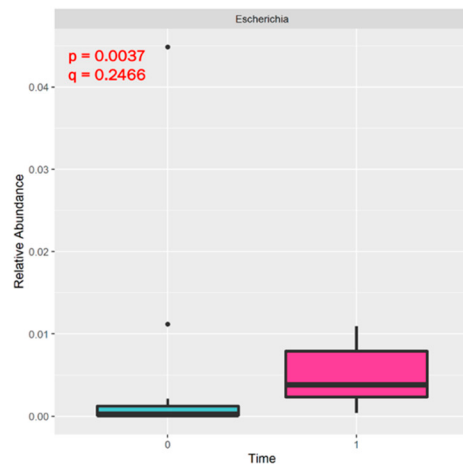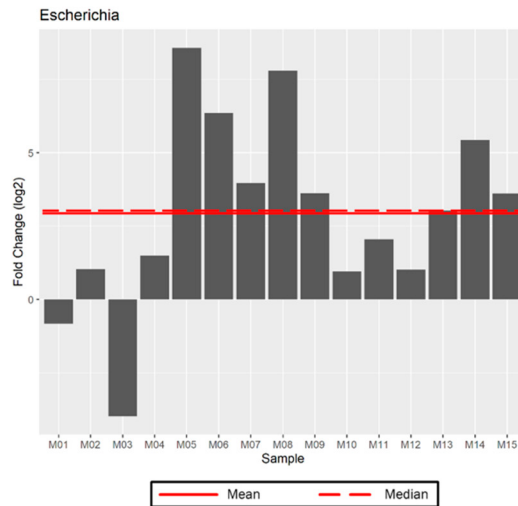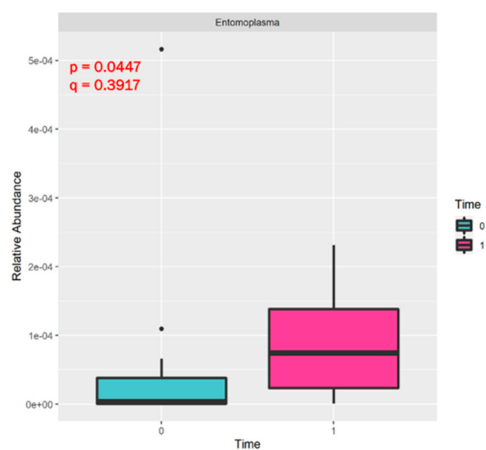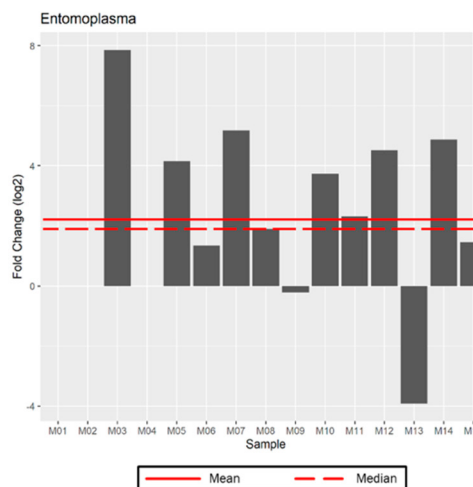

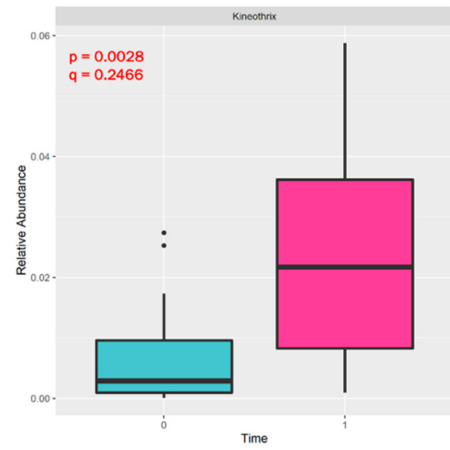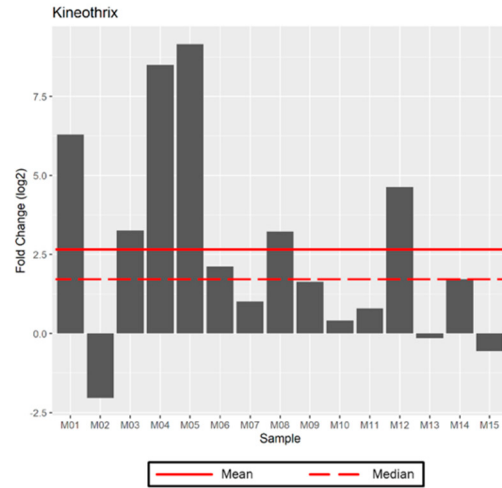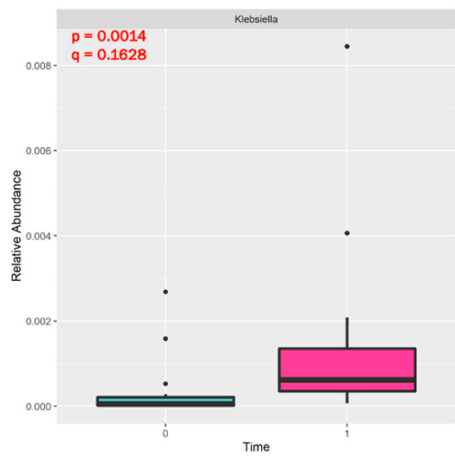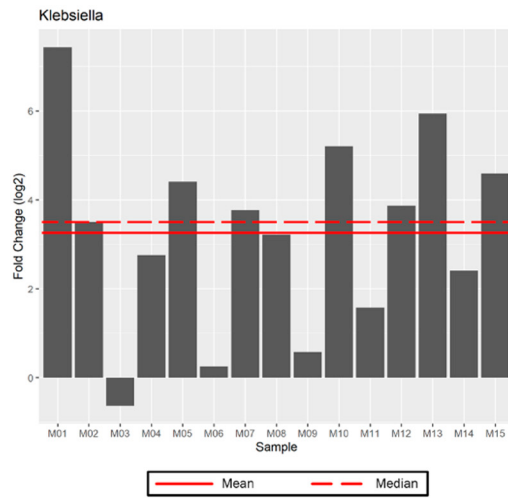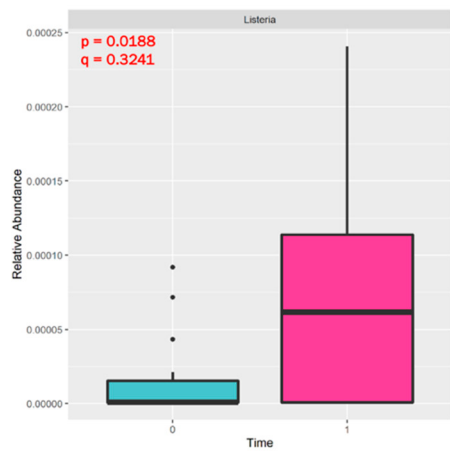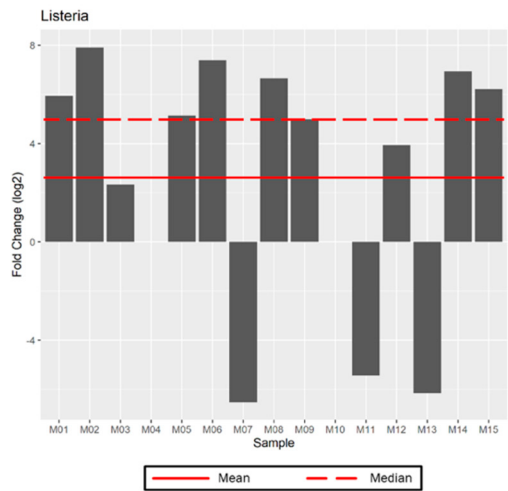

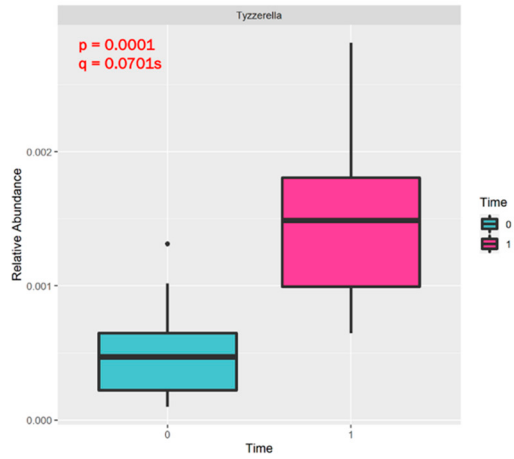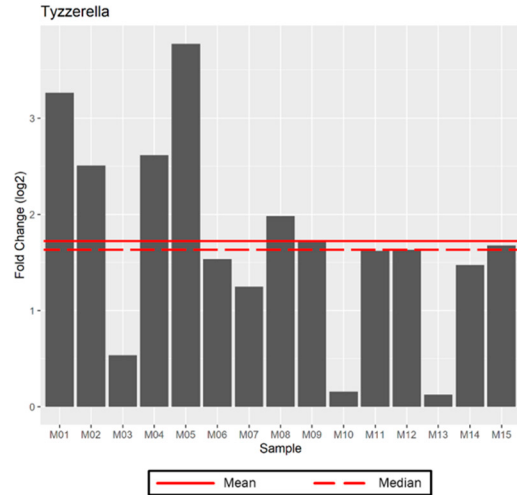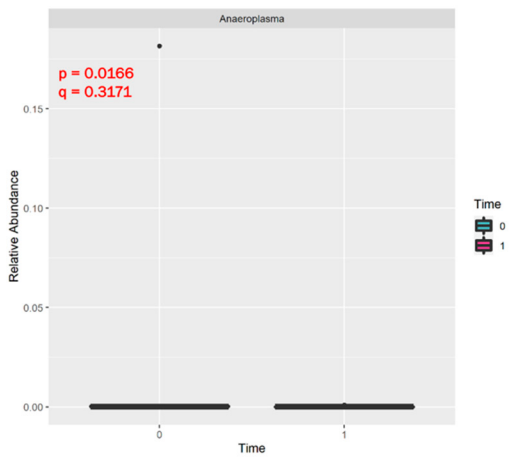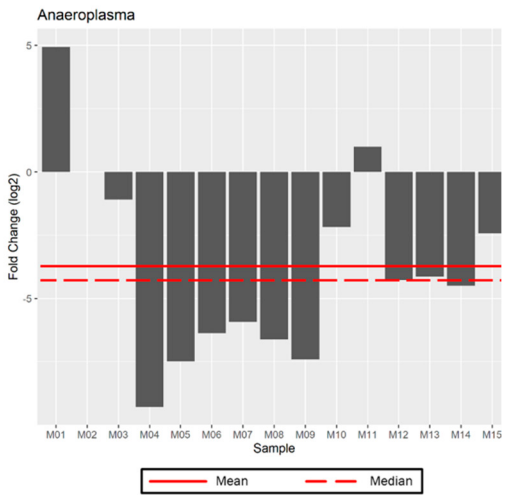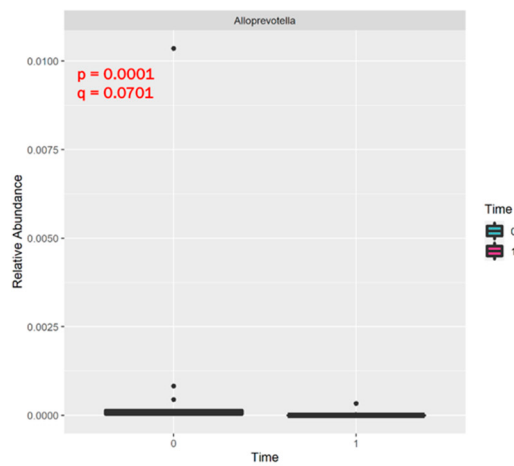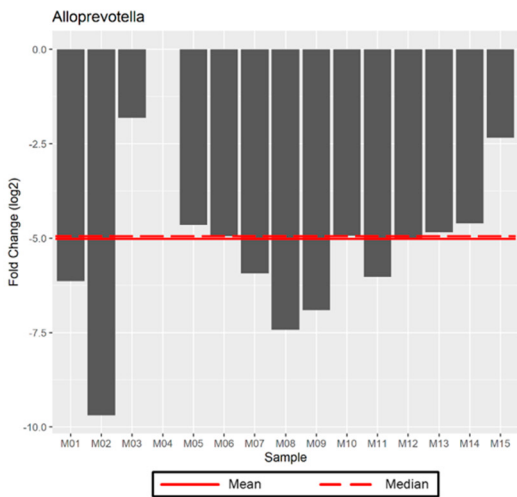

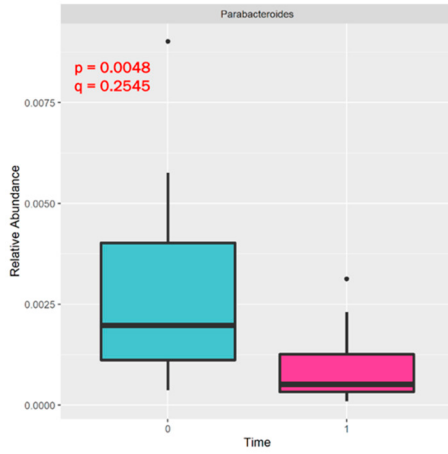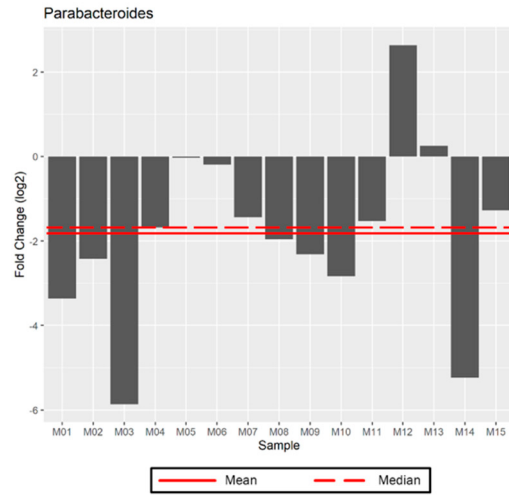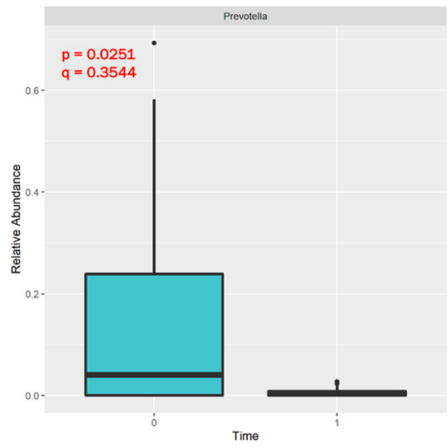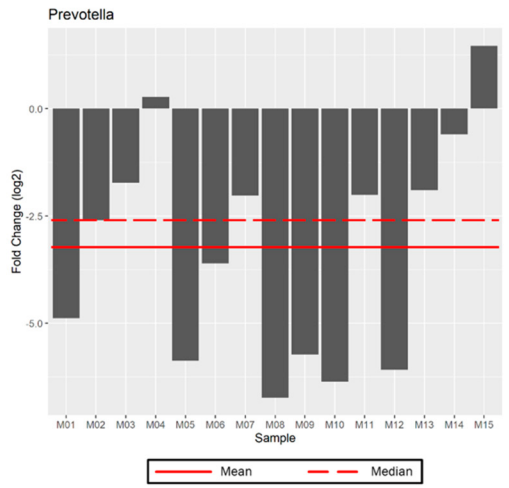

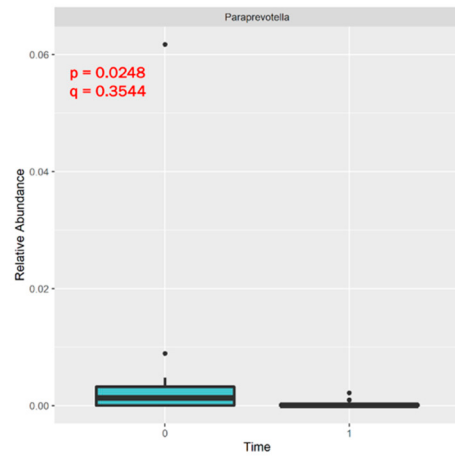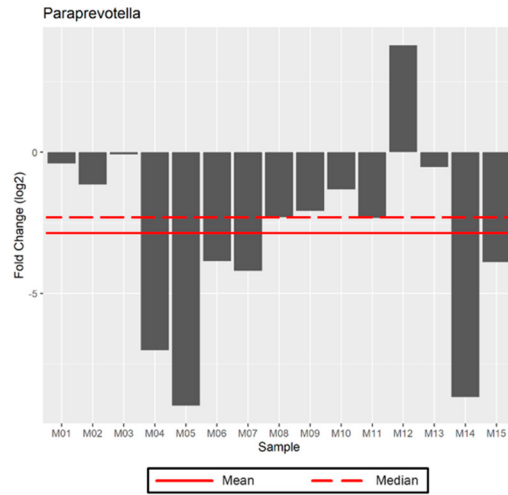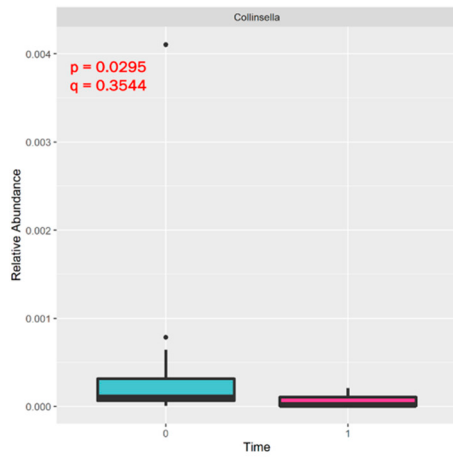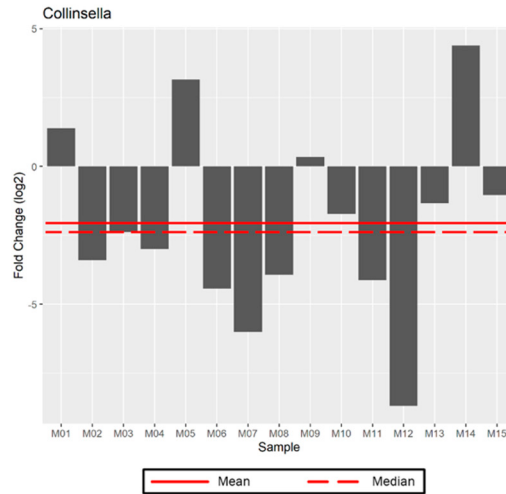

**Supplementary Figure S2.** Genus with 2-fold or more variation

Supplement: Supplementary file 1 [file nutrients-16-04374-s001.zip › Supplementary Figure S2.pdf]
